# Efficacy of Colonoscopic Decompression in the Management of Ogilvie Syndrome: A Systematic Review

**DOI:** 10.7759/cureus.92815

**Published:** 2025-09-20

**Authors:** Shahab Ud Din Zia, Pranav S Shukla, Roopa Chalasani, Sojeong Mun, Gilles Van de Vel

**Affiliations:** 1 Medicine and Surgery, Pak International Medical College, Peshawar, PAK; 2 Medicine, Grant Medical College and Sir JJ Group of Hospitals, Mumbai, IND; 3 Research, Wake Forest Institute for Regenerative Medicine, Winston Salem, USA; 4 Physical Medicine and Rehabilitation, Hallym University College of Medicine, Chuncheon, KOR; 5 Hospital Medicine, King's Mill Hospital, Sutton-in-Ashfield, GBR

**Keywords:** acute colonic pseudo-obstruction, clinical outcomes, colonoscopic decompression, colonoscopy, gastroentrology, neostigmine, ogilvie syndrome, systemic review

## Abstract

Ogilvie syndrome, also known as acute colonic pseudo-obstruction (ACPO), is characterized by massive dilation of the colon without any mechanical obstruction. This condition primarily affects elderly patients, particularly those who are hospitalized or have multiple comorbidities. The pathophysiology remains poorly understood, but it is believed to stem from an imbalance in the autonomic nervous system that disrupts normal colonic motility. ACPO can lead to significant complications, including colonic perforation, if not managed appropriately. Several treatment modalities are currently used for ACPO. Initially, conservative management is deployed, followed by endoscopic or medical intervention with neostigmine. Surgical colectomy or colostomy is offered as a last resort when other interventions either fail or are contraindicated. Following the Preferred Reporting Items for Systematic Reviews and Meta-Analyses (PRISMA) 2020 guidelines, we conducted a systematic literature search in PubMed/Medline, Europe PMC, Cochrane Library, Science Direct, EBSCO Open Dissertation, and ClinicalTrial.gov. A total of 12 studies were included in our review, with sample sizes ranging from one to 138 participants (a total of 387). The eligibility criteria focused on adults aged 18 and older who were diagnosed with Ogilvie syndrome, dilated colon with no physical bowel obstruction on imaging studies, and underwent colonoscopic decompression as a treatment. Colonoscopic decompression presents a promising intervention for Ogilvie syndrome, showing superior efficacy to neostigmine; however, relapses are common. The administration of polyethylene glycol (PEG) reduces relapses and improves the effectiveness of colonoscopic decompression. Future research should focus on larger randomized controlled trials (RCTs) to validate these findings, address existing gaps, and explore long-term outcomes.

## Introduction and background

Ogilvie syndrome, also known as acute colonic pseudo-obstruction (ACPO), was first described in 1948 as massive colonic dilation in the absence of any mechanical obstruction [1]. The exact pathophysiology of Ogilvie syndrome remains unclear, but it is believed to arise from the disruption of the autonomic innervation of the colon, likely sympathetic overdrive, reduced parasympathetic activity, or a combination of the two. ACPO is a rare condition, predominantly observed in elderly patients who are hospitalized or have multiple comorbidities [2]. Studies indicate an incidence of approximately one case per 1,000 inpatient admissions [3]. A large retrospective review of 400 cases identified the most common associated conditions with ACPO in surgical patients: trauma (11.3%), obstetrics and gynecology (9.8%), infection (10%), cardiac (10%), and neurological conditions (9.3%) [4].

Despite its rarity, Ogilvie syndrome significantly impacts the U.S. healthcare system. A large-scale study analyzing cases from 1998 to 2011 found that total hospital charges for managing ACPO nearly tripled, increasing from $25,316 ± 41,034 to $66,471 ± 110,209 (P < 0.0001). This study also reported a decrease in annualized deaths from 631 (9.4%) to 538 (6.4%), yielding an incidence of 9.2 versus 6.7 deaths per 100,000 patients per year (P < 0.0001). The overall mortality rate during this period was 7.7% [3].

Diagnosis of ACPO primarily involves radiological investigations to rule out mechanical obstruction. CT scan with contrast is the investigation of choice [2]. A serious complication of ACPO is colonic perforation, with estimates suggesting perforation risks of 0%, 7%, and 23% for cecal diameters of <12 cm, 12 to 14 cm, and >14 cm, respectively [4]. Early detection of ACPO is crucial because it can reduce complications and alleviate the financial burden on the healthcare system.

Management of Ogilvie syndrome according to the American Society for Gastroentrointestinal Endoscopy involves conservative treatment as the initial approach, followed by medical intervention with neostigmine and then colonoscopic decompression as secondary management. If abdominal distention persists, surgical options such as a surgically placed cecostomy tube, percutaneous cecostomy, and subtotal colectomy are employed [5].

Recent evidence indicates that colonoscopic decompression, first utilized in the 1970s, may be a more effective alternative to conservative management and medical intervention with neostigmine [6,7]. It is the preferred invasive procedure for patients with significant cecal distention (>10 cm) of prolonged duration (>3-4 days) that does not improve after 24-48 hours of supportive therapy, particularly when contraindications to or failures of pharmacological therapy exist. Initial success rates for decompression are estimated to range between 61% and 95% [7]. However, comprehensive studies evaluating the safety and efficacy of colonoscopic decompression are limited. Our knowledge of the safety and efficacy of colonoscopic decompression as a therapy for ACPO is limited by the lack of RCTs and inadequate information on long-term results.

This systematic review addresses these gaps while gaining a clearer understanding of its role in clinical practice. The outcomes of interest include the resolution of colonic distention and improvement of clinical symptoms reported by patients and/or imaging studies. In addition, the review will evaluate the incidence of complications related to the procedure and the necessity for subsequent surgical interventions.

By examining both efficacy and safety, this systematic review will contribute to the development of evidence-based guidelines for treating Ogilvie syndrome.

## Review

Methods

Study Design

The purpose of this systematic study is to assess the efficacy of colonoscopic decompression compared to conservative therapy in adult patients with Ogilvie syndrome. To guarantee a thorough and open methodology, the review adheres to the Preferred Reporting Items for Systematic Reviews and Meta-Analyses (PRISMA) 2020 criteria [8]. The primary research question guiding the review was: What is the efficacy of colonoscopic decompression compared to conservative treatment in adult patients with Ogilvie syndrome in terms of clinical outcomes and complications? The main goal is to develop an understanding of the clinical outcomes of colonoscopic decompression and its complications in order to gain important information about how effective this treatment option is.

PICO Framework

The review is structured around the Patient, Intervention, Comparison, and Outcome (PICO) framework, which outlines the patient population, intervention, comparison, and outcomes of interest. The patient population includes adult patients aged 18 years and older who have been diagnosed with Ogilvie syndrome. The intervention under consideration is colonoscopic decompression, while the comparison involves conservative treatment approaches, such as supportive care and observation. The outcomes of interest include the resolution of colonic distention, as measured by imaging studies, and improvement in clinical symptoms. In addition, the review will assess the incidence of complications related to the procedure and the necessity for surgical intervention.

Eligibility Criteria

Inclusion criteria for this review specified that only individuals aged 18 years and older were eligible for inclusion. The review considered original studies that aligned with the PICO framework, which encompassed randomized controlled trials (RCTs), controlled clinical trials (CCTs), observational studies, case series, and case reports. Only completed trials with reported results were included, and the focus was on moderate to high-quality studies that may have presented some concerns but maintained a low risk of bias. Only research conducted in the English language was included.

The exclusion criteria entailed several specific parameters. We did exclude studies involving individuals under the age of 18, as well as those involving animals or failing to align with the PICO framework. Furthermore, non-original research has not been considered, including reviews, abstracts, posters, editorials, commentaries, and opinions. Unfinished or ongoing trials without published results, including preprints, were also excluded, as were completed studies that did not report results. Articles in languages other than English were deemed ineligible.

Data Collection

An extensive literature search was conducted across multiple databases, including PubMed, Medical Literature Analysis and Retrieval System Online (MEDLINE), Cochrane Library (CENTRAL), Europe PubMed Central (PMC), Elton B. Stephens Company (EBSCO) Open Dissertations, ScienceDirect, and ClinicalTrial.gov. The search strategy was designed to find relevant studies on the efficacy of colonoscopic decompression in managing ACPO. The search commenced on November 26, 2024, and concluded on November 27, 2024. We used different search terms to find relevant articles. For example, in PubMed/MEDLINE, the search included terms such as “efficacy”, “colonoscopic decompression”, and “Ogilvie’s syndrome”, among others. Similar terms were adapted for other databases to fit their specific search functionalities (Table 1).

**Table 1:** Search strategy

Database	Search strategy	Number of studies before / after filters	Filters used
Cochrane Library (CENTRAL)	( Efficacy OR "effectiveness" OR "success" OR "outcomes" OR "therapeutic effect" OR "clinical benefit" OR "treatment efficacy" OR "Treatment Outcome*") AND ((“Colonoscopic decompression” OR "colonic decompression" OR "endoscopic decompression" OR "colonoscopy for decompression" OR "endoscopic colonic intervention") AND (“Ogilvie’s syndrome” OR "acute colonic pseudo-obstruction" OR "colonic pseudo-obstruction" OR "pseudo-obstruction of the colon" OR "Ogilvie's disease")	4/4	Cochrane Trials, English
EBSCO Open Dissertations	( Efficacy OR "effectiveness" OR "success" OR "outcomes" OR "therapeutic effect" OR "clinical benefit" OR "treatment efficacy" OR "Treatment Outcome*") AND ((“Colonoscopic decompression” OR "colonic decompression" OR "endoscopic decompression" OR "colonoscopy for decompression" OR "endoscopic colonic intervention") AND (“Ogilvie’s syndrome” OR "acute colonic pseudo-obstruction" OR "colonic pseudo-obstruction" OR "pseudo-obstruction of the colon" OR "Ogilvie's disease")	0/0	Dissertations, English
PubMed/MEDLINE	( Efficacy OR "effectiveness" OR "success" OR "outcomes" OR "therapeutic effect" OR "clinical benefit" OR "treatment efficacy" OR "Treatment Outcome"[Mesh] OR "Outcome Assessment, Health Care"[Mesh]) AND (“Colonoscopic decompression” OR "colonic decompression" OR "endoscopic decompression" OR "colonoscopy for decompression" OR "endoscopic colonic intervention") AND (“Ogilvie’s syndrome” OR "acute colonic pseudo-obstruction" OR "colonic pseudo-obstruction" OR "pseudo-obstruction of the colon" OR "Ogilvie's disease" OR "Colonic Pseudo-Obstruction"[Mesh]) OR "Colonic Pseudo-Obstruction/therapy"[Mesh])	509/43	Full free text clinical trial, clinical control trial, randomized control trial, case reports, observational studies
Europe PMC	( Efficacy OR "effectiveness" OR "success" OR "outcomes" OR "therapeutic effect" OR "clinical benefit" OR "treatment efficacy" OR "Treatment Outcome*") AND ((“Colonoscopic decompression” OR "colonic decompression" OR "endoscopic decompression" OR "colonoscopy for decompression" OR "endoscopic colonic intervention") AND (“Ogilvie’s syndrome” OR "acute colonic pseudo-obstruction" OR "colonic pseudo-obstruction" OR "pseudo-obstruction of the colon" OR "Ogilvie's disease")	144/86	Research article, full text in Europe PMC, link to full free text,
ScienceDirect	( Efficacy OR "effectiveness" OR "outcomes") AND (“Colonoscopic decompression” OR "colonic decompression" OR "Endoscopic Decompression") AND (“Ogilvie’s syndrome” OR "acute colonic pseudo-obstruction" OR "colonic pseudo-obstruction" )	282/6	Research article, case reports, open access, English
Clinical Trial.gov	( Efficacy OR "effectiveness" OR "success" OR "outcomes" OR "therapeutic effect" OR "clinical benefit" OR "treatment efficacy" OR "Treatment Outcome*") AND ((“Colonoscopic decompression” OR "colonic decompression" OR "endoscopic decompression" OR "colonoscopy for decompression" OR "endoscopic colonic intervention") AND (“Ogilvie’s syndrome” OR "acute colonic pseudo-obstruction" OR "colonic pseudo-obstruction" OR "pseudo-obstruction of the colon" OR "Ogilvie's disease")	0/0	

Study Selection and Screening

The collected studies from all databases and registers were sent to the Rayyan application, an artificial intelligence platform, for the purpose of removing duplicates and screening by title and abstract [9]. This process facilitated the application of the eligibility criteria established for the review. Each study was assessed for relevance, ensuring that only those that met the predefined inclusion criteria progressed to full-text review. The app Rayyan allowed for efficient collaboration among the review team and enabled them to independently evaluate studies and reach a consensus on which articles should be included in the final analysis. This systematic approach aimed to enhance the rigor and reliability of the review process.

Quality Assessment

The quality of the included studies was assessed using appropriate tools such as the Cochrane Risk of Bias Tool 2 (RoB 2) for RCTs studies [10], the Newcastle-Ottawa Scale (NOS) for observational studies [11], and the Joanna Briggs Institute (JBI) checklist specified for case reports and case series [12,13]. These assessments helped categorize the studies based on their quality and allowed for a focused analysis of those with moderate to high quality. By adhering to these protocols, the review aims to provide reliable conclusions regarding the efficacy of colonoscopic decompression in managing Ogilvie syndrome.

Data Synthesis and Analysis

Data synthesis and analysis were carried out using both narrative summaries and tables. The narrative summary provided an overview of the findings, highlighting the efficacy and safety of colonoscopic decompression compared to conservative treatment for Ogilvie syndrome. This approach highlighted key outcomes and contextualized the clinical implications of these results.

A summary table was created to present the characteristics of each included study, providing a detailed overview of study design, sample size, and intervention-specific outcome measures and key findings. This tabular format facilitated quick comparisons and assessments of overall trends.

Results

Study Selection

**Figure 1:**
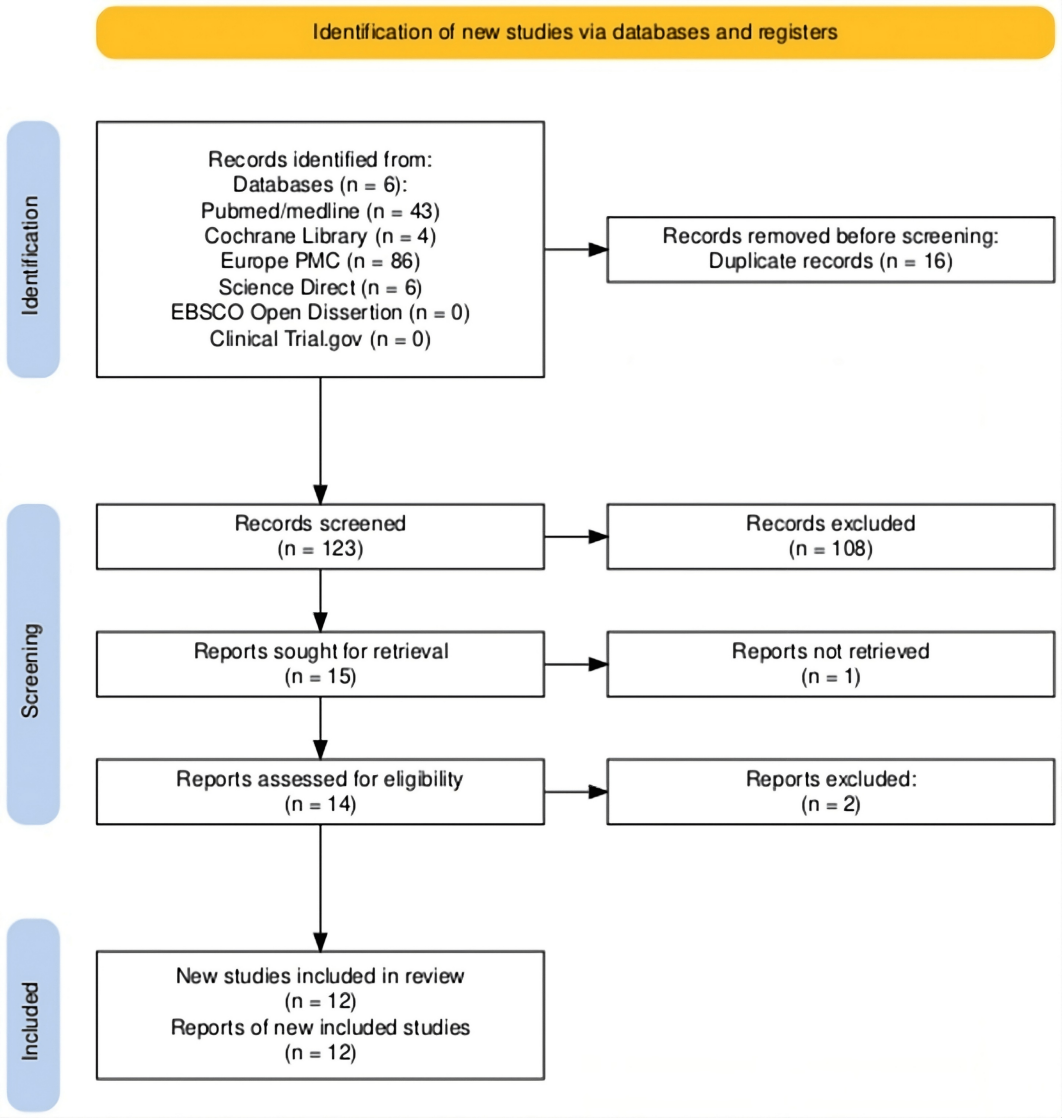
Detailed PRISMA 2020 flow diagram PRISMA: Preferred Reporting Items for Systematic Reviews and Meta-Analyses

Upon completing the search strategy, a total of 139 studies were identified across eight databases. After eliminating duplicates using the Rayyan application and applying the established inclusion and exclusion criteria [9], 15 studies were selected for full-text review. Ultimately, 12 studies fulfilled the eligibility criteria and were included in this systematic review, as depicted in the PRISMA 2020 flow diagram (see Figure 1).

*Results of Quality Appraisal* 

In alignment with the PRISMA guidelines [8], we performed a quality assessment of the included studies. Table 2 presents the quality assessment of observational studies based on the NOS scale [11]. For studies to be included in future reviews using the NOS assessment, they must achieve a minimum score of 7 out of 9. In our review, all included studies scored at least 8, meeting this criterion for inclusion.

**Table 2:** Newcastle-Ottawa scale assessment of observational studies Note: Newcastle-Ottawa permits four stars (*) for selection, two for comparability, and three for outcome. The total scores range between 0 and 9.

Author/year	Study design	Selection	Comparability	Outcomes	Overall
Haj et al., 2018 [14]	Retrospective cohort	****	**	***	9/9 Good quality
Liu et al., 2021 [6]	Retrospective cohort	****	*	***	8/9 Good quality
Gulisano et al., 2023 [15]	Retrospective cohort	***	**	***	8/9 Good quality
Zhao et al., 2017 [16]	Prospective cohort	****	*	***	8/9 Good quality
Fausel & Goff, 1985 [17]	Retrospective cohort	****	*	***	8/9 Good quality
Peker et al., 2017 [18]	Retrospective cohort	****	**	***	9/9 Good quality

Table 3 presents the risk of bias assessment for the RCT study using the Cochrane RoB 2 tool [10]. Each domain indicates a low risk of bias for all assessed domains, suggesting that the results are reliable and valid.

**Table 3:** Cochrane risk of bias 2 tool for randomised clinical trials The domains are as follows: (1) bias due to the randomization process, (2) deviation from intended intervention, (3) missing outcome data, (4) measurement of outcomes, (5) selection of the reported result.

Author	Domain 1	Domain 2	Domain 3	Domain 4	Domain 5	Overall
Sgouros et al., 2006 [19]	Low risk of bias	Low risk of bias	Low risk of bias	Low risk of bias	Low risk of bias	Low risk of bias

Table 4 details the quality appraisal of case reports using the JBI quality appraisal checklist [12]. All assessed case reports received high scores, indicating good quality and thorough reporting of their methodologies and outcomes.

**Table 4:** Quality appraisal of case reports using the JBI quality appraisal checklist JBI: Joanna Briggs Institute; Q: question ✅ - Yes; ⛔ - No; ❓- Unclear; ❎- Not applicable

Author/year	Q 1	Q 2	Q 3	Q 4	Q 5	Q 6	Q 7	Q 8	Overall appraisal / decision
Numan et al., 2023 [20]	✅	✅	✅	✅	✅	✅	✅	✅	8/8 Included
Liu et al., 2020 [21]	✅	✅	✅	✅	✅	✅	✅	✅	8/8 Included
Kim et al., 2013 [22]	✅	✅	✅	✅	✅	✅	✅	✅	8/8 Included
Hsu et al., 2011 [23]	✅	✅	✅	✅	✅	✅	✅	✅	8/8 Included

Table 5 provides the quality appraisal of case series using the JBI quality appraisal checklist [13]. The assessed case series also demonstrated good quality, indicating thorough methodology and reporting of outcomes.

**Table 5:** Quality appraisal of case series using the JBI quality appraisal checklist JBI: Joanna Briggs Institute; Q: question ✅ - Yes;  ⛔ - No; ❓- Unclear;  ❎- Not applicable

		Tenofsky et al., 2000 [24]
Q 1	Were there clear criteria for inclusion in the case series?	✅
Q 2	Was the condition measured in a standard, reliable way for all participants included in the case series?	✅
Q 3	Were valid methods used for identification of the condition for all participants included in the case series?	✅
Q 4	Did the case series have consecutive inclusion of participants?	✅
Q 5	Did the case series have complete inclusion of participants?	✅
Q 6	Was there clear reporting of the demographics of the participants in the study?	✅
Q 7	Was there clear reporting of clinical information of the participants?	✅
Q 8	Were the outcomes or follow up results of cases clearly reported?	✅
Q 9	Was there clear reporting of the presenting site(s)/clinic(s) demographic information?	✅
Q 10	Was statistical analysis appropriate?	✅
	Overall Appraisal	Good Quality

Characteristics of the Included Studies

The included studies on Ogilvie syndrome consisted of one RCT, one case series, one prospective cohort study, five retrospective cohort studies, and four case reports, with sample sizes ranging from one to 138 participants. Interventions primarily included neostigmine, which was utilized for its effectiveness in promoting colonic motility and colonoscopic decompression, often performed after neostigmine when initial treatments were ineffective. Supportive care, such as nasogastric tubes and fluid resuscitation, was also part of the management strategies (Table 6).

**Table 6:** Summary of included studies IV: intravenous; PEG: polyethylglycol; ACPO: acute colonic pseudo-obstruction; PEC: percutaneous endoscopic colostomy; ACPO-T: acute colonic pseudo-obstruction-thickness (gut wall); ACPO-NT: acute colonic pseudo-obstruction-no thickness (gut wall)

Study reference	Study design	Sample size	Intervention	Comparison	Key findings
Haj et al., 2018 [14]	Retrospective cohort	37	Interventional management, including neostigmine, colonoscopy, and surgery if needed	Conservative management, including observation, rectal tube, nasogastric tube, fluid resuscitation, and electrolyte correction	No significant difference in bowel dilation (12.0 cm vs. 13.0 cm; P = 0.21). Similar comorbidity scores (CCI), higher complication rate in the interventional group (61% vs. 21%; P < 0.01). No difference in the overall length of stay or resolution time between groups. Inpatient mortality was low (5.4% conservative vs. 5.6% interventional). Conservative management is associated with fewer complications and similar outcomes to interventional approaches.
Liu et al., 2021 [6]	Retrospective cohort	42	First supportive care; if ineffective: second neostigmine, third decompressive colonoscopy	No formal control group	Both neostigmine and decompressive colonoscopy were effective for treating ACPO, with response rates of 86.67% and 95.8%, respectively (P = 0.390). Adverse events were rare; one case of transient bradycardia in the neostigmine group and one bowel perforation occurred two weeks post-colonoscopy.
Gulisano et al., 2023 [15]	Retrospective	5	Initial management: Bowel rest, rectal and nasogastric tube placement, fluid and electrolyte replacement. Subsequent interventions: Endoscopy for decompression in two patients, neostigmine and rifaximin administered to some.	No control group	ACPO developed a mean of 33.8 days after COVID-19 symptom onset, lasting an average of 24.6 days. All patients had ICU admissions; one patient died due to septic shock. The remaining patients resolved symptoms, without surgery. Resolved after initial management: four patients. Resolved after endoscopic decompression: one patient.
Zhao et al., 2017 [16]	Prospective cohort	138	Colonoscopic decompression in ACPO-NT	Colonoscopic decompression in ACPO-T	Colonoscopic decompression had better results in the ACPO-NT group than in the ACPO-T group, although with no significant difference (75% vs. 42.86%, p = 0.318). Patients in the ACPO-NT group had significantly higher efficacy in conservative treatment (62.50% vs. 24.44%, p < 0.001). Neostigmine efficacy was only 17.64% in the ACPO-T group, which was significantly lower than that in the ACPO-NT group (77.78%, p < 0.001).
Fausel and Goff, 1985 [17]	Retrospective cohort	27	Initial conservative management, flexible colonoscopy for non-responders, and surgical intervention (cecostomy) if needed	No control group	Initial conservative measures were effective in treating acute idiopathic colonic pseudo-obstruction. Colonoscopic decompression was successful in patients with cecal diameters ≥12 cm who did not respond to conservative treatment. Five patients required surgical intervention (cecostomy), and the overall mortality rate was 30%, with only one death directly attributable to pseudo-obstruction.
Peker et al., 2017 [18]	Retrospective cohort	68	Group 1: Colonoscopic decompression after conservative treatment. Group 2: Neostigmine treatment followed by colonoscopic decompression if there was a poor response.	Group 1 served as a control for the effectiveness of neostigmine in Group 2.	Response to the first intervention: Group 1: 15/31 (48.4%) had a good response. Group 2: 31/37 (83.8%) had a good response. Statistical significance: p = 0.002 (significant). Overall response: Group 1: 26/31 (83.9%), total response. Group 2: 33/37 (89.2%), total response. Statistical Significance: p = 0.722 (not significant). Hospital stay: Group 1: 9.48 ± 6.80 days. Group 2: 8.59 ± 4.75 days. Statistical Significance: p = 0.743 (not significant). Recurrence at one month: Group 1: 4/31 (12.9%), Group 2: 4/34 (11.8%).
Sgouros et al., 2006 [19]	Randomized clinical trial	30	First: neostigmine (2 mg IV) or endoscopic decompression for the initial treatment. Second: PEG (29.5 g daily for seven days) after resolution	First: initial treatment with neostigmine or endoscopic decompression. Second: placebo for PEG.	33.3% of patients in the placebo group experienced recurrent cecal dilation compared to none in the PEG group (p = 0.04). PEG therapy led to significant increases in stool (p = 0.001) and flatus evacuations (p = 0.032). Significant decrease in caecum, ascending and transverse colon diameters, and abdominal circumference (p = 0.017, 0.018, 0.014, and 0.008, respectively).
Numan et al., 2023 [20]	Case report	1	First: medical management. Second: colonoscopic compression, performed twice. Third: cecostomy tube placement. Fourth: PEC tube placement.	Not applicable	Colonic distension: Initially measured at 14 cm, improved to 9 cm after conservative treatment but worsened to 10.7 cm upon readmission. Post-procedure outcomes: Colonoscopic decompression: Performed twice without sustained resolution. Cecostomy tube placement: Failed to relieve distension, which reached 22 cm. Successful placement of the PEC tube led to improvement in colonic distension to less than 10 cm. Follow-up at six weeks showed no abdominal distension, and the tube remained patent. No acute complications reported from the PEC tube placement.
Liu et al., 2020 [21]	Case report	1	First: initial management with nasogastric tube, docusate, and fleet enema. Second: decompressive colonoscopy on postoperative days 8 and 10 performed twice.	Not applicable	Ogilvie syndrome diagnosed post-VP shunt; conservative management followed by two decompressive colonoscopies; the second decompression improved colonic distension and relieved abdominal pain, but the patient had a prolonged hospital stay due to recurrence of symptoms before achieving complete resolution.
Kim et al., 2013 [22]	Case report	1	Repetitive colonoscopic decompression; subtotal colectomy at 21 weeks gestation	Not applicable	IV administration of neostigmine was not effective. Three attempts of decompression before subtotal colectomy. Post-surgery, stool passage normalized, and pregnancy continued to full term.
Hsu et al., 2011 [23]	Case report	1	First: nasogastric decompression and discontinuation of oral intake. Second: colonoscopic decompression followed by intravenous neostigmine.	Not applicable	Initial conservative management failed; colonoscopic decompression followed by neostigmine led to full recovery.
Tenofsky et al., 2000 [24]	Case series	36	Initial management: supportive care (observation, fluid resuscitation). Subsequent Interventions: neostigmine for pharmacological treatment, decompressive procedures (colonoscopy, rectal tube) as needed.	No control group	Conservative treatment was successful in 52.8% of cases (n = 19). Twenty colonoscopic decompressions were performed on 13 patients, with an overall success rate of 77% (n = 10). Of the three patients in whom colonoscopic decompression failed, two died, and one required operation. Five of the 36 patients required surgical intervention, with a mortality rate of 60% (n = 3).

Discussion

The management of Ogilvie syndrome (ACPO) remains a complex challenge in clinical practice, particularly regarding the efficacy of colonoscopic decompression compared to conservative treatment. This discussion synthesizes the findings from various case reports, case series, observational studies, and RCTs to provide a comprehensive overview of the current evidence surrounding Ogilvie syndrome.

Evidence From Case Reports and Case Series

Case reports and series have been instrumental in highlighting the clinical outcomes associated with colonoscopic decompression. For example, Tenofsky et al. (2000) conducted a case series involving 36 patients diagnosed with Ogilvie syndrome, revealing that 24 of these patients were men, with an average age of 68.9 years [24]. The study found that conservative treatment was successful in approximately 52.8% of cases, while colonoscopic decompression had a success rate of 77%. However, it is noteworthy that 46% of patients who initially responded to decompression experienced symptom recurrence, necessitating further interventions [24].

In the case report by Hsu et al., a 76-year-old patient who failed conservative treatment was successfully managed by colonoscopic decompression followed by neostigmine without any further complications [23]. By contrast, Liu et al. (2020) reported a case of a 76-year-old patient who developed Ogilvie syndrome after undergoing ventriculoperitoneal shunt placement for normal pressure hydrocephalus. This patient required multiple decompression attempts after conservative management failed, ultimately achieving symptom resolution. This variability in outcomes highlights the individual nature of Ogilvie syndrome [21].

Kim et al. (2013) presented a case involving a pregnant patient in her early 30s who experienced chronic intestinal pseudo-obstruction precipitated by pregnancy. She could not pass stool for over four weeks and required repetitive colonoscopic decompression before ultimately undergoing a total colectomy [22]. This case emphasizes the potential need for surgical intervention in refractory cases, especially in unique populations such as pregnant women.

The recent case report by Numan et al. (2023) highlighted a 68-year-old patient with acute colonic pseudo-obstruction following a Clostridium difficile infection. This patient did not respond to medical treatment or endoscopic decompression, leading to the successful placement of a percutaneous endoscopic colostomy (PEC) tube [20]. This showcases PEC tube placement as a viable alternative for patients at high risk for surgical management, indicating that while colonoscopic decompression remains a primary intervention, alternative approaches may prove beneficial for specific patient populations.

These case reports collectively suggest that while colonoscopic decompression can be effective, it is not universally successful, and outcomes can vary significantly based on individual patient factors and the underlying etiology of Ogilvie syndrome [20,21,22,23,24]. The risk of adverse outcomes, such as colonic perforation and recurrence of symptoms, further complicates the clinical decision-making process.

Evidence From Cohort Studies

Cohort studies have provided critical insights into management and outcomes of Ogilvie syndrome, providing both similarities and differences in patient presentations and treatment responses across various contexts. For instance, Gulisano et al. (2023) focused on patients hospitalized with severe COVID-19 who developed ACPO and identified five individuals who required admission to the intensive care unit (ICU). The symptoms of ACPO in this cohort emerged after a mean of 33.8 days from the onset of COVID-19, highlighting the syndrome's potential as serious. The treatment primarily involved colonic decompression through rectal and nasogastric tubes with most patients resolving their gastrointestinal symptoms without surgical intervention [15]. This study underscores the urgent need to recognize ACPO early in seriously ill patients, emphasising the need for prompt and effective management to mitigate complications.

By contrast, the work of Fausel and Goff (1985) presents a broader analysis of acute idiopathic colonic pseudo-obstruction over four years, encompassing 35 episodes in 27 patients. This cohort, with a mean age of 61 years, included individuals with severe underlying conditions such as cancer and respiratory failure. Their results showed that flexible colonoscopy in non-responders after initial conservative treatments was safe and effective [17]. These findings imply that favourable results can be obtained with a nonoperative technique. While Gulisano et al. emphasize the critical nature of ACPO in the context of COVID-19, Fausel and Goff highlight the potential for successful conservative management in less acute scenarios [15,17].

Further complicating the picture, Zhao et al. (2017) differentiated between ACPO patients with and without acute gut wall thickening. Their study found that those with thickening exhibited significantly higher Acute Physiology and Chronic Health Evaluation (APACHE II) and Sequential Organ Failure Assessment scores (SOFA scores), indicating a more severe clinical state and a higher 28-day mortality rate [16]. These findings suggest that patients with gut wall thickening may need more aggressive management strategies, highlighting the variability in patient presentation and the necessity for individualised treatment approaches.

In addition, Haj et al. (2018) reviewed 37 patients and found no significant differences in outcomes between conservative and interventional management strategies, although complications were more frequent in those undergoing interventions [14]. This reinforces the idea that while interventional strategies are often emphasized, conservative management may be equally effective, particularly in less severe cases.

Moreover, Liu et al. (2021) analyzed 46 cases of ACPO and found that both neostigmine and colonoscopic decompression were effective, with response rates of 86.7% and 95.8%, respectively [7]. This suggests that while both treatments are viable options, colonoscopic decompression may offer superior outcomes in specific. This aligns with findings from Peker et al. (2016), which indicated that colonoscopic decompression was more successful as an initial therapy compared to neostigmine [18], further supporting the need for careful consideration of treatment options based on individual patient circumstances.

Evidence From RCTs

The RCT by Sgouros et al. (2006) provides strong evidence for the efficacy of colonoscopic decompression in patients with Ogilvie syndrome. The study showed that patients receiving colonoscopic decompression had significantly better outcomes, including reduced colonic dilation and improved stool passage, compared to those on placebo [19]. Involving 30 patients with colonic dilation, the trial randomized participants to receive polyethylene glycol (PEG) or a placebo daily for seven days after treatment with neostigmine or endoscopic decompression. Neostigmine achieved an 88% success rate, while colonoscopic decompression was successful in all 8 patients without any complications. The placebo group had a 33.3% relapse rate, compared to 0% in the PEG group (p = 0.04). PEG also significantly enhanced stool and flatus evacuations and reduced colonic diameters and abdominal circumference [19]. These results underscore PEG's potential to improve colonoscopic decompression outcomes and minimize relapse rates.

Strengths and Limitations of the Included Studies

The included studies in this review show several strengths that enhance the credibility of the findings. The range of study designs, including RCTs, cohort studies, and case reports, provides a multifaceted view of the efficacy of colonoscopic decompression in managing Ogilvie syndrome. In addition, the majority of studies were rated as moderate to high quality with low risk of bias, which benefits the reliability of conclusions drawn. The clinical relevance of the outcomes assessed, such as symptom resolution and complication rates, underlines the practical implications of the research.

Despite these strengths, the included studies also have notable limitations. Many of the RCTs featured small sample sizes, which may limit the statistical power and generalizability. The variability in treatment protocols and definitions of success further complicates direct comparisons and the synthesis of findings. In addition, several studies were observational, introducing potential biases that limit the causal inferences regarding treatment efficacy. Unfortunately, many studies lack sufficient long follow-up periods to evaluate long-term outcomes such as symptom recurrence or complications.

Strengths and Limitations of Our Review

The reliability and relevance of our systematic review are enhanced by a number of its strengths. The wide search strategy applied across multiple databases produced a variety of relevant content. When assessing the effectiveness of colonoscopic decompression in comparison to conservative treatment, the organised PICO framework made it easier to stay focused and clear. In addition, the comprehensive assessment of the quality of the included studies supports the validity of our conclusions and enables a more in-depth analysis of the data.

However, there are certain limitations to our review. Non-randomized studies can be more biased than RCTs, despite the fact that their inclusion offers valuable information. Another limitation of our review was the inclusion criteria, which restricted the analysis to English-language articles. This may introduce linguistic bias, which could limit the review's inclusiveness and skew its overall findings. Furthermore, some papers might not have been acquired due to accessibility issues. In addition, these findings may not have external validity because they may not be applicable to all patient populations, especially those with distinct clinical presentations or comorbidities not covered in the included research.

Future Research Directions

Future research on the efficacy of colonoscopic decompression in managing Ogilvie syndrome should focus on conducting more RCT and CCT While the majority of studies available are case reports and cohort studies, these types of studies often lack the rigor needed to draw definitive conclusions. The limited number of high-quality RCTs underlines the need for robust research designs that can provide clearer insights into the effectiveness of colonoscopic decompression compared to conservative treatment.

In addition, many of the observational studies included in our review suffer from small sample sizes, which limits the power of their findings. Future studies would hope to recruit larger cohorts to enhance the statistical validity of the results. Further longer follow-up periods are essential to evaluate long-term outcomes such as recurrence rates and complications because they are crucial for understanding the full impact of the intervention.

Important to future research is addressing potential confounding factors. Variations in patient demographics, underlying health conditions, and the severity of colonic distension can significantly influence treatment outcomes. By controlling for these factors, researchers can improve the reliability of their findings and provide a more nuanced understanding of the efficacy of colonoscopic decompression.

Further future studies should consider implementing subgroup analyses to identify specific patient populations that may benefit the most from colonoscopic decompression. This approach will facilitate the development of personalized treatment strategies tailored towards individual patient needs. Finally, multicenter collaborations should be encouraged to pool resources and expertise in order to enable the collection of larger datasets that can enhance the generalizability of findings across different healthcare settings. By prioritizing these research directions, we can gain a deeper understanding of Ogilvie Syndrome and improve treatment strategies for patients.

## Conclusions

Colonoscopic decompression has demonstrated notable efficacy in managing Ogilvie syndrome. This intervention can lead to substantial improvements in clinical outcomes, including the resolution of colonic distention and symptom relief. While general safety is important, the successful outcomes and risk of complications can depend on the expertise of the physician performing a colonoscopy. The existing literature also underscores that prompt diagnosis and early intervention can mitigate the risk of adverse complications. In addition, colonoscopic decompression has shown superior efficacy compared to neostigmine alone, and a combination of both interventions yields even better results. The administration of PEG can prevent relapses and occurrences, increasing the effectiveness of colonoscopic decompression. Despite the potential for complications, colonoscopic decompression remains a rapid and effective method of achieving symptom relief. 

Future research should prioritize well-designed RCTs to strengthen the evidence base surrounding colonoscopic decompression. Larger sample sizes and longer follow-up periods will be essential to assess long-term outcomes such as recurrence rates, complications, and quality of life. In addition, multicenter collaboration would allow for exploring the efficacy of this intervention across diverse patient populations and would help refine treatment protocols and enhance personalized care strategies for Ogilvie’s syndrome patients. By addressing these research gaps, we can better manage management approaches and ultimately optimize patient outcomes.
